# Plasma biomarkers of neurodegeneration in the brain (UCH‐L1 and NfL) become exponentially higher with age from early childhood, and treatment of Alzheimer’s disease participants with granulocyte‐macrophage colony‐stimulating factor (GM‐CSF) lowers age‐associated levels of plasma UCH‐L1 by six decades

**DOI:** 10.1002/alz.092726

**Published:** 2025-01-09

**Authors:** Stefan H Sillau, Christina M Coughlan, Md. Mahiuddin Ahmed, Kavita V Nair, Paula Araya, Matthew D. Galbraith, Brianne M Bettcher, Joaquin M. Espinosa, Heidi J Chial, Neill Epperson, Timothy D. Boyd, Huntington Potter

**Affiliations:** ^1^ University of Colorado Department of Neurology and Alzheimer's and Cognition Center, Aurora, CO USA; ^2^ University of Colorado Alzheimer's and Cognition Center, Aurora, CO USA; ^3^ Department of Neurology, and Department of Clinical Pharmacy, University of Colorado, Aurora, CO USA; ^4^ Linda Crnic Institute for Down Syndrome, University of Colorado Anschutz Medical Campus, Aurora, CO USA; ^5^ University of Colorado Alzheimer's and Cognition Center/Universiyt of Colorado Anschutz Medical Campus, Aurora, CO USA; ^6^ Linda Crinic Institute for Down Syndrome, Universiyt of Colorado Anschutz Medical Campus, Aurora, CO USA; ^7^ Department of Neurology, University of Colorado Alzheimer's and Cognition Center, and the Linda Crnic Institute for Down Syndrome, University of Colorado, Anschutz Medical Campus, Aurora, CO USA; ^8^ University of Colorado Anschutz Medical Campus, Aurora, CO USA; ^9^ University of Colorado Alzheimer's and Cognition Center, University of Colorado Anschutz Medical Campus, Aurora, CO USA

## Abstract

**Background:**

Increasing age is the greatest risk factor for age‐associated cognitive decline (AACD) and, especially in females, for developing Alzheimer’s disease (AD). Mechanisms underlying this connection are unknown, but neuronal loss and brain atrophy accompany aging and AD and likely contribute to cognitive deficits. There are currently no means to measure neuronal cell death during life and no means to prevent it. Inflammation is also correlated with AD pathogenesis and aging (‘inflammaging’), but whether inflammation causes and/or is a response to neurodegeneration is unknown.

Treatment of mouse models of AD with GM‐CSF reverses amyloid deposition and normalizes cognition, and GM‐CSF treatment improves cognition in aged wild‐type mice. A published double‐blind placebo‐controlled phase II trial showed that three weeks of treatment with recombinant human GM‐CSF (sargramostim) in mild‐to‐moderate AD participants improved cognition (MMSE) and blood biomarkers of amyloid, Tau, and neurodegeneration. Together, these results suggest that GM‐CSF may slow or halt the neuronal damage in aging and/or AD that causes cognitive deficits.

**Method:**

Plasma concentrations of ubiquitin C‐terminal hydrolase‐L1, UCH‐L1, a measure of neuronal cell loss, neurofilament light (NfL), a measure of axonal damage, and GFAP, a measure of inflammation/astrogliosis, were assessed cross‐sectionally in 317 healthy control participants between age 2 and 85 using the Quanterix SIMOA platform. The resulting age curves were compared to the results from the previously published clinical trial of sargramostim/GM‐CSF in participants with mild‐to‐moderate AD.

**Result:**

The concentrations of human plasma proteins released from dying/damaged neurons (UCH‐L1 and NfL) showed an exponential increase from age 2‐85 and rise more in females. Plasma GFAP concentrations are exponentially higher after age 40, following neurodegeneration. Treatment of AD participants in the GM‐CSF/sargramostim trial halted neuronal cell death, based on the reduction in plasma UCH‐L1 concentrations to levels equivalent to those of five‐year‐old healthy controls. The ability of GM‐CSF treatment to reduce neuronal apoptosis was confirmed in the hippocampi of aged TgF344‐AD rats, a model recapitulates all human AD brain neuropathology.

**Conclusion:**

These findings suggest that age‐associated exponential increases in neurodegeneration may underlie the contribution of aging to cognitive decline, including in AD, and that GM‐CSF/sargramostim treatment may halt these effects.

